# CdS/rGO Nanocomposite for Sensitive Electrochemical Detection of Copper Ions

**DOI:** 10.3390/s26216729

**Published:** 2026-10-24

**Authors:** Muhammad Sajid Shafiq, Fabian O. Romero-Soto, Martín F. Jiménez-Moreno, Amritha Kadathamana, Masoud Madadelahi, Marc J. Madou, Josué Amilcar Aguilar Martínez, Rudra Kumar

**Affiliations:** 1 School of Engineering and Sciences, Tecnologico de Monterrey, Av. Eugenio Garza Sada 2501 Sur, Monterrey 64849, NL, Mexicofabianrs@tec.mx (F.O.R.-S.); martin.jimenez@tec.mx (M.F.J.-M.); masoud.m@tec.mx (M.M.); mmadou@tec.mx (M.J.M.); 2 Centro de Investigación e Innovación en Ingeniería Aeronáutica (CIIIA), Facultad de Ingeniería Mecánica y Eléctrica, Universidad Autónoma de Nuevo León, Carretera a Salinas Victoria km. 2.3, Apodaca 66600, NL, Mexico; amritha.kadathamanax@uanl.edu.mx (A.K.); josue.aguilarmrt@uanl.edu.mx (J.A.A.M.); 3 Department of Mechanical and Aerospace Engineering, University of California, Irvine, CA 92697, USA; 4 Facultad de Ingeniería Mecánica y Eléctrica, Universidad Autónoma de Nuevo León, Av. Universidad, Cd. Universitaria, San Nicolás de los Garza 66455, NL, Mexico

**Keywords:** CdS/rGO nanocomposite, electrochemical sensor, differential pulse anodic stripping voltammetry, copper ion sensing, real sample analysis

## Abstract

A highly sensitive and selective electrochemical sensor for Cu^2+^ ions was fabricated by modifying a glassy carbon electrode (GCE) with a CdS/rGO nanocomposite. The structural and morphological characteristics of the synthesized materials were thoroughly investigated using Fourier-transform infrared spectroscopy (FTIR), X-ray diffraction (XRD), field-emission scanning electron microscopy (FESEM), energy-dispersive X-ray spectroscopy (EDX), X-ray photoelectron spectroscopy (XPS), and Raman spectroscopy. These analyses confirmed the synthesis of the CdS/rGO nanocomposite via a hydrothermal synthesis route. The electrochemical performance of the modified electrode was systematically evaluated using cyclic voltammetry (CV), differential pulse anodic stripping voltammetry (DPASV), and electrochemical impedance spectroscopy (EIS). The CdS/rGO-modified electrode exhibited enhanced electrocatalytic activity, with well-defined anodic and cathodic peak currents that demonstrated a linear relationship with the square root of the scan rate over the range of 50–250 mV s^−1^. Under optimized conditions, the sensor demonstrated appropriate analytical performance over a Cu^2+^ concentration range of 10–100 nM, with a remarkably low limit of detection (LOD) of 2.83 nM. Furthermore, the sensor exhibited good selectivity toward Cu^2+^ in the presence of various interfering ions, along with good reproducibility and long-term operational stability. Its practical applicability was further demonstrated through the successful determination of Cu^2+^ ions in water samples and complex matrices, yielding satisfactory recovery values. These results highlight the potential of the CdS/rGO-based sensor as a reliable and sensitive platform for the practical electrochemical detection of Cu^2+^ ions.

## 1. Introduction

In recent decades, the rapid expansion of agricultural and industrial activities has resulted in the release of various toxic heavy metal ions into the ecosystem. These contaminants can accumulate in water bodies and food sources, raising concerns regarding their impacts on ecosystems and human health. Exposure to heavy metals at concentrations above permissible levels has been associated with several adverse health effects, including lung and bladder cancers, skin lesions, and other metal-specific disorders [1,2,3]. To minimize human exposure, the World Health Organization (WHO) provides recommended guideline values for heavy metal concentrations in drinking water, including Pb^2+^ (0.0483 µM), Cd^2+^ (0.0267 µM), Hg^2+^ (0.029 µM), As^3+^ (0.133 µM), and Cu^2+^ (47.21 µM) (Guidelines for Drinking Water Quality, 2015) [4]. Although many heavy metals are toxic at elevated concentrations, certain trace metals are essential for maintaining normal physiological functions. These elements contribute to metabolic regulation, the synthesis and functioning of metalloenzymes and metalloproteins, and various transcriptional processes [5,6]. Among them, copper (Cu^2+^) is an essential micronutrient that plays a crucial role in several physiological processes, including erythropoiesis and connective tissue formation. As one of the most abundant essential trace elements, alongside iron (Fe^2+^/Fe^3+^) and zinc (Zn^2+^), copper serves as an important catalytic cofactor in numerous enzymatic reactions. However, excessive exposure to Cu^2+^ can disrupt cellular homeostasis and promote the generation of reactive oxygen species (ROS). In biological systems, Cu^2+^ can undergo redox cycling through its reduction to Cu^+^ (Cu^2+^ + e^−^ → Cu^+^), leading to the generation of highly reactive species, including hydroxyl radicals (•OH). These reactive species can interact with biomolecules such as lipids, proteins, and DNA, leading to oxidative damage and increasing the risk of kidney and liver dysfunction, neurological disorders, and gastrointestinal disturbances [7,8,9,10].

Given these concerns, there is a growing need for reliable, sensitive, and convenient sensing platforms for the monitoring of Cu^2+^ ions in food and water samples. Conventional analytical techniques for Cu^2+^ detection include atomic absorption spectroscopy [11], UV-visible spectroscopy [12], fluorescence spectrometry [13,14], colorimetric analysis [15,16], and immunoassays [17]. Although these techniques offer high sensitivity and accuracy, their practical application is often constrained by sophisticated instrumentation, lengthy analysis procedures, high operational costs, and the requirement for skilled personnel. Moreover, their limited portability makes them less suitable for rapid and real-time monitoring [18]. In this context, electrochemical sensing techniques have emerged as promising alternatives owing to their high sensitivity, excellent reproducibility, operational simplicity, portability, rapid response, and cost-effectiveness [19,20,21,22]. In particular, electrochemical sensors based on chalcogenide nanomaterials have attracted considerable attention for the detection of heavy metal ions because of their favorable electrical, optical, and electrochemical properties. Group II–VI chalcogenides, including CdSe, ZnS, CuS, and CdS, exhibit distinctive physicochemical characteristics, such as tunable band gaps, high absorption coefficients, and sharp absorption edges [23,24]. Among these materials, cadmium sulfide (CdS) has received significant attention because of its tunable band gap, excellent electrochemical properties, high surface activity, and strong affinity toward target analytes. Furthermore, the incorporation of CdS into electrode architectures can facilitate electron-transfer processes and enhance the electrochemical current response, thereby improving the sensitivity and overall analytical performance of the sensor.

Despite these advantages, the practical application of CdS can be hindered by rapid electron-hole recombination and limited structural stability [25]. To address these limitations, various strategies, including controlled doping, morphological engineering, and optimization of synthesis conditions, have been investigated. The integration of CdS with conductive supporting materials, such as carbon nanotubes (CNTs) and reduced graphene oxide (rGO), has also emerged as an effective approach for enhancing its electrochemical performance and analytical response [26]. In particular, coupling CdS nanoparticles with rGO can significantly improve electrical conductivity while providing a large specific surface area, thereby facilitating efficient electron transport and increasing the availability of active sites [27,28]. Furthermore, the incorporation of rGO can enhance the stability of the sensing platform by promoting charge transfer, suppressing CdS degradation, and facilitating the uniform dispersion of CdS nanoparticles within a highly conductive network. These synergistic effects can improve signal reproducibility and long-term operational stability. Thus, the CdS/rGO hybrid architecture combines the advantageous properties of both components, offering enhanced sensitivity, selectivity, and stability for electrochemical sensing applications [29]. Several studies have demonstrated the potential of modified electrodes for the electrochemical detection of Cu^2+^ ions. For example, Senthil et al. reported a PBS/GNS/GCE-based electrochemical sensor for Cu^2+^ determination, exhibiting a linear response over the concentration range of 1 × 10^−8^ to 1 × 10^−4^ M with a detection limit of 1 × 10^−8^ [30]. Wang et al. fabricated an rGO-gold nanoparticle-carbonized resin nanosphere composite for Cu^2+^ sensing, with a detection limit of 6 μg L^−1^ [31]. Similarly, Mabrouk et al. fabricated a ZnO@EDTA-Ag-modified GCE and evaluated its performance using differential pulse voltammetry (DPV), obtaining a broad linear range of 0.007–10 μM and a low LOD of 7 nM [32].

From this perspective, CdS/rGO nanocomposites represent a promising yet relatively underexplored material platform for electrochemical sensing, particularly for the sensitive detection of hazardous Cu^2+^ ions. In the present study, a CdS/rGO nanocomposite was developed as the active sensing platform for electrochemical determination of Cu^2+^ ions. The analytical performance of the modified electrode was investigated using differential pulse anodic stripping voltammetry (DPASV), with particular emphasis on Cu^2+^ sensitivity and selectivity. The CdS/rGO composite was prepared through a simple one-step hydrothermal synthesis, yielding a robust electrode material with favorable electrical conductivity and electrochemical stability. The interactive combination of CdS and rGO provides abundant electroactive sites and efficient electron-transfer pathways, thereby significantly enhancing the electrochemical response toward Cu^2+^ ions. Importantly, the proposed sensor exhibits high sensitivity and a low detection limit compared with several previously reported Cu^2+^ sensors. These results highlight the potential of the CdS/rGO-modified electrode as a reliable and sensitive platform for the practical monitoring of Cu^2+^ ions in environmental and other complex samples.

## 2. Results and Discussion

### 2.1. FESEM

**Figure 1:**
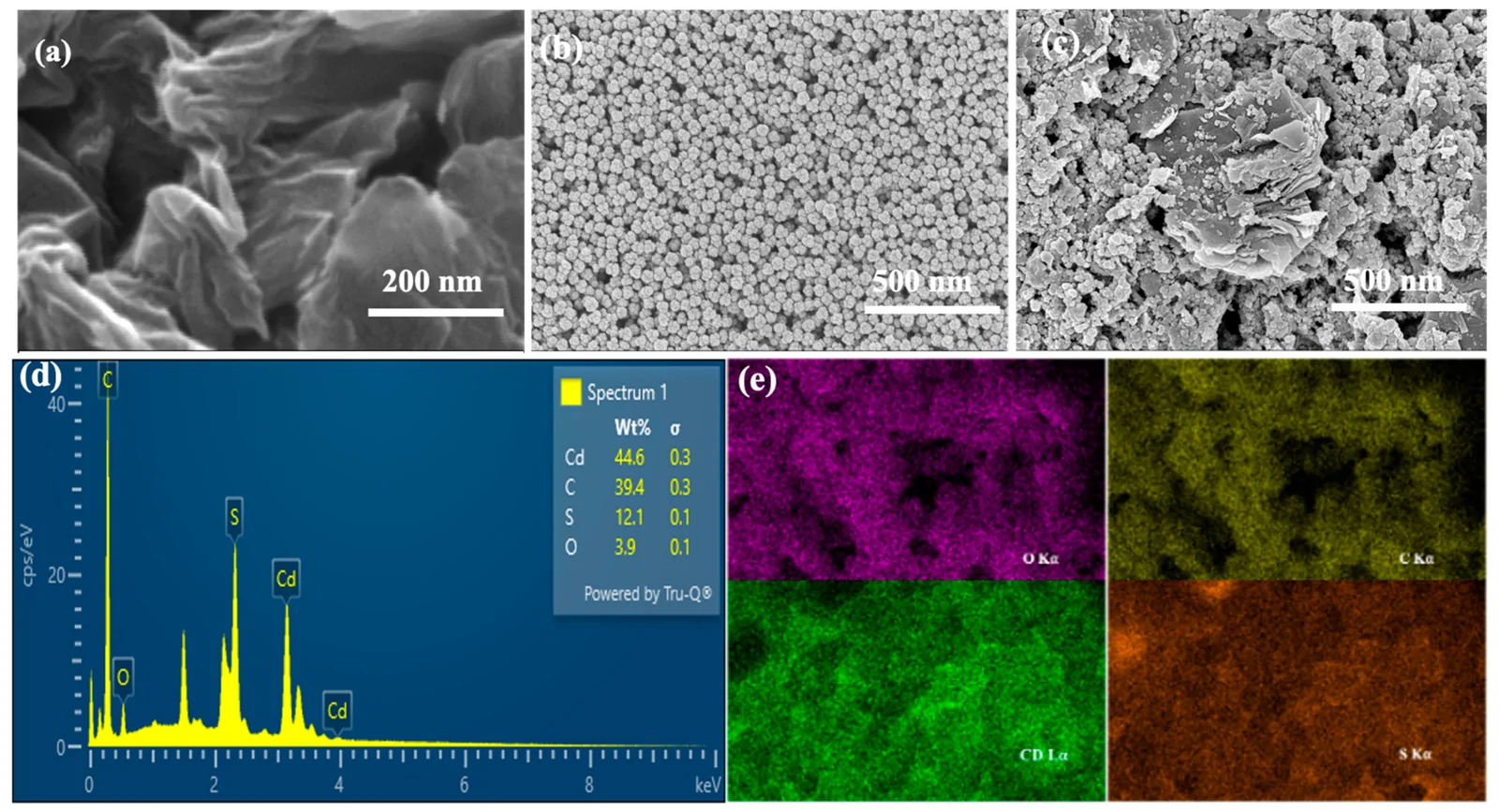
FESEM images of (**a**) GO, (**b**) CdS, and (**c**) CdS/rGO nanocomposite; (**d**) EDX spectrum and (**e**) elemental mapping of CdS/rGO nanocomposite.

The FESEM images of the synthesized GO, CdS, and CdS/rGO composites are presented in Figure 1. The FESEM image of GO (Figure 1a) reveals a characteristic wrinkled, sheet-like morphology with several overlapping layers [33]. The lateral dimensions of the GO sheets range from approximately 200 nm to 2 μm. At higher magnification, the GO surface appears relatively smooth, with distinct folds and corrugations. These morphological features are characteristic of the flexible and highly corrugated structure of GO nanosheets and can significantly influence their surface properties and electrical behavior, making them attractive for electrochemical sensing applications [34]. Moreover, the wrinkled and curled morphology provides a larger accessible surface area and facilitates the formation of efficient electron-transfer pathways, which can contribute to enhanced electrochemical sensing performance. The FESEM image of the CdS nanoparticles (Figure 1b) shows a predominantly spherical morphology, with particle sizes ranging from approximately 500 nm to 2 μm. The relatively uniform distribution of CdS nanoparticles provides abundant accessible active sites and can facilitate charge transfer, thereby contributing to the improved electrochemical performance of the sensing electrode [35].

As shown in Figure 1c, the CdS nanoparticles are uniformly distributed over the rGO nanosheets, with particle sizes ranging from approximately 500 nm to 2 μm. This homogeneous distribution is highly beneficial for electrochemical sensing and catalytic applications, as it maximizes the contact between the CdS nanoparticles and the rGO substrate, thereby facilitating efficient charge transfer and enhancing electrochemical activity. Furthermore, the EDX spectrum shown in Figure 1d confirms the elemental composition of the CdS/rGO nanocomposite, with characteristic signals corresponding to Cd, S, C, and O. The elemental mapping results presented in Figure 1e further demonstrate the homogeneous spatial distribution of Cd, S, C, and O throughout the nanocomposite. These results confirm the successful integration and uniform dispersion of CdS nanoparticles within the rGO matrix.

### 2.2. Structural and Morphological Characterization

**Figure 2:**
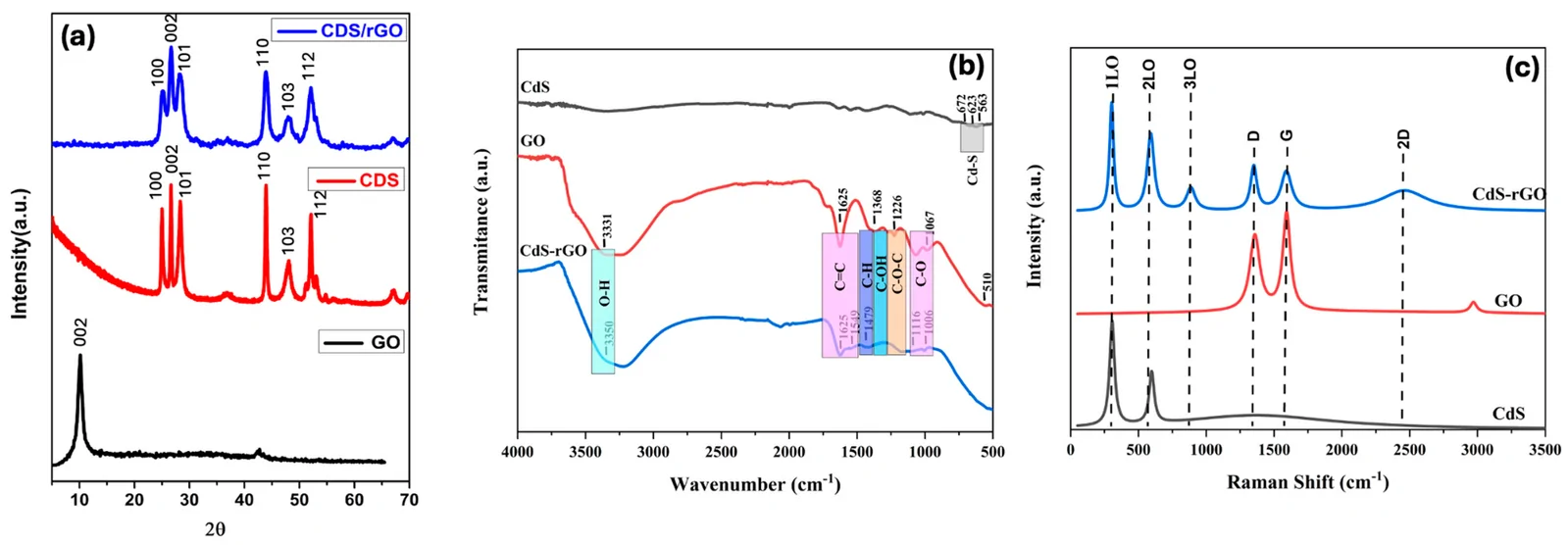
(**a**–**c**) Comprehensive XRD, FTIR, and Raman analysis of the CdS/rGO nanocomposite.

Figure 2a presents the XRD patterns of GO, CdS nanoparticles, and the CdS/rGO nanocomposite. The GO pattern exhibits a characteristic sharp diffraction peak at 2θ = 10.5°, which can be indexed to the (002) plane and is associated with the increased interlayer spacing resulting from the incorporation of oxygen-containing functional groups compared with pristine graphite. The XRD pattern of the CdS nanoparticles displays prominent diffraction peaks at 2θ = 24.7°, 26.4°, 28.3°, 36.5°, 43.6°, 47.7°, and 51.9°, corresponding to the (100), (002), (101), (102), (110), (103), and (112) crystallographic planes, respectively. These reflections are consistent with the hexagonal crystal structure of CdS and agree well with the standard JCPDS reference card No. 02-0549, confirming the formation of phase-pure CdS. The XRD pattern of the CdS/rGO nanocomposite exhibits characteristic diffraction features associated with both CdS and rGO, confirming the successful formation of the hybrid nanocomposite [36]. The coexistence of the characteristic reflections from both components indicates the successful integration of CdS nanoparticles with the rGO framework. This structural combination may facilitate charge-transfer processes, which could contribute to the enhanced electrochemical performance of the CdS/rGO-based sensing platform.

Figure 2b presents the FTIR spectra of GO, CdS, and the CdS/rGO nanocomposite. The FTIR spectrum of GO exhibits several characteristic absorption bands associated with oxygen-containing functional groups, providing evidence of its surface chemical composition. A broad and intense band centered at approximately 3331 cm^−1^ is attributed to the O-H stretching vibration, confirming the presence of hydroxyl groups on the GO surface [37]. The absorption band observed at around 1067 cm^−1^ can be assigned to C-O stretching vibrations, primarily associated with epoxy and other oxygen-containing functional groups. The band at approximately 1625 cm^−1^ is attributed to the O-H bending vibration of adsorbed water molecules and the skeletal vibration of sp^2^-hybridized C=C bonds within the graphene framework [38]. In comparison, the FTIR spectrum of the CdS nanoparticles exhibits characteristic absorption bands in the 500–700 cm^−1^ region, which can be assigned to Cd-S stretching vibrations, confirming the formation of the CdS phase. For the CdS/rGO nanocomposite, the significant attenuation or disappearance of the oxygen-containing functional-group bands observed in GO indicates the effective reduction of GO to rGO during the hydrothermal process. In addition, a distinct band at approximately 1549 cm^−1^ is observed, which can be attributed to the skeletal vibrations of the sp^2^-carbon framework of rGO [39]. These FTIR results further support the successful formation of the CdS/rGO nanocomposite.

Figure 2c presents the Raman spectra of GO, CdS, and the CdS/rGO nanocomposite, providing further evidence for the successful integration of rGO within the composite matrix. The Raman spectrum of GO exhibits D and G bands at 1372 cm^−1^ and 1594 cm^−1^, respectively. The D band is associated with structural defects and disorder within the carbon framework, whereas the G band arises from the in-plane vibrational modes of sp2-hybridized carbon atoms [40]. In contrast, the Raman spectrum of CdS nanoparticles displays characteristic peaks at approximately 300 cm^−1^ and 600 cm^−1^, which correspond to the first- and second-order longitudinal optical (1LO and 2LO) phonon modes, respectively. For the CdS/rGO nanocomposite, the D and G bands are observed at approximately 1366 cm^−1^ and 1586 cm^−1^, respectively. The slight shifts in these bands toward lower wavenumbers compared with those of GO indicate changes in the carbon structure associated with the reduction of GO during the hydrothermal synthesis process. Furthermore, the increased I_D_/I_G_ intensity ratio of the CdS/rGO nanocomposite relative to GO provides additional evidence of the reduction of GO and the formation of rGO [41]. The appearance of an additional band at approximately 2395 cm^−1^, attributed to the 2D band, further supports the structural transformation of GO into rGO within the CdS/rGO nanocomposite.

The XPS survey spectra presented in Figure S1a further confirm the successful integration of CdS with rGO, with characteristic signals corresponding to Cd, S, C, and O detected in the CdS/rGO nanocomposite. The high-resolution Cd 3d spectrum exhibits two prominent peaks at 405.0 and 411.7 eV (spin–orbit splitting of 6.8 eV), which can be assigned to the Cd 3d_5/2_ and Cd 3d_3/2_ states, respectively, confirming the presence of Cd^2+^ species in the CdS lattice [42]. A weaker doublet with its Cd 3d_5/2_ line at 406.2 eV (18% of the Cd 3d area) indicates a minor contribution of oxidized Cd species, most likely surface hydroxide or sulfate (see Supplementary Information (Figure S1b)). Similarly, the high-resolution S 2p spectrum (Figure S1c) was fitted with spin–orbit doublets, each consisting of an S 2p_3/2_ and an S 2p_1/2_ line separated by 1.21–1.27 eV. The dominant doublet, with the S 2p_3/2_ and S 2p_1/2_ lines at 161.3 and 162.6 eV (67% of the S 2p area), confirms the presence of sulfide (S^2−^) in the CdS lattice [43]. A second doublet (S 2p_3/2_ at 162.3 eV, 26%) is attributed to thiol-type C–S sulfur from residual thiourea-derived species, and a minor doublet at 168.7 eV (7%) to oxidized sulfur. The feature near 162 eV therefore contains both the sulfide S 2p_1/2_ line and the thiol S 2p_3/2_ line. The fitting model and the parameters of all components are given in the Supplementary Information (Figure S1c and Table S2). The deconvoluted O 1s spectrum can be resolved into three distinct components (Figure S1e). The component at higher binding energy (532.8 eV, 22% of the O 1s area) can be attributed to C-O species associated with hydroxyl and other oxygen-containing functional groups on the rGO surface, whereas the dominant component at 531.5 eV (76%) is assigned primarily to carbonyl (C=O) groups. A weak component at 530.0 eV (2%) can be attributed to metal–oxygen species, possibly arising from the partial surface oxidation of CdS and the formation of Cd–O bonds [44]. The C 1s spectrum exhibits a dominant peak characteristic of sp^2^-hybridized graphitic carbon (284.4 eV, 64% of the C 1s area), accompanied by contributions from sp^3^-hybridized and defect-related carbon species (Figure S1d). Additional components at higher binding energies can be attributed to oxygen-containing functional groups, including hydroxyls, epoxy, carbonyl, and carboxyl species. These results further confirm the presence of the rGO framework and its successful integration with CdS in the nanocomposite. The fitting procedure and the parameters of all Cd 3d, S 2p, C 1s, and O 1s components are given in the Supplementary Information (Tables S1–S4).

### 2.3. Electrochemical Characterization and Optimization 

**Figure 3:**
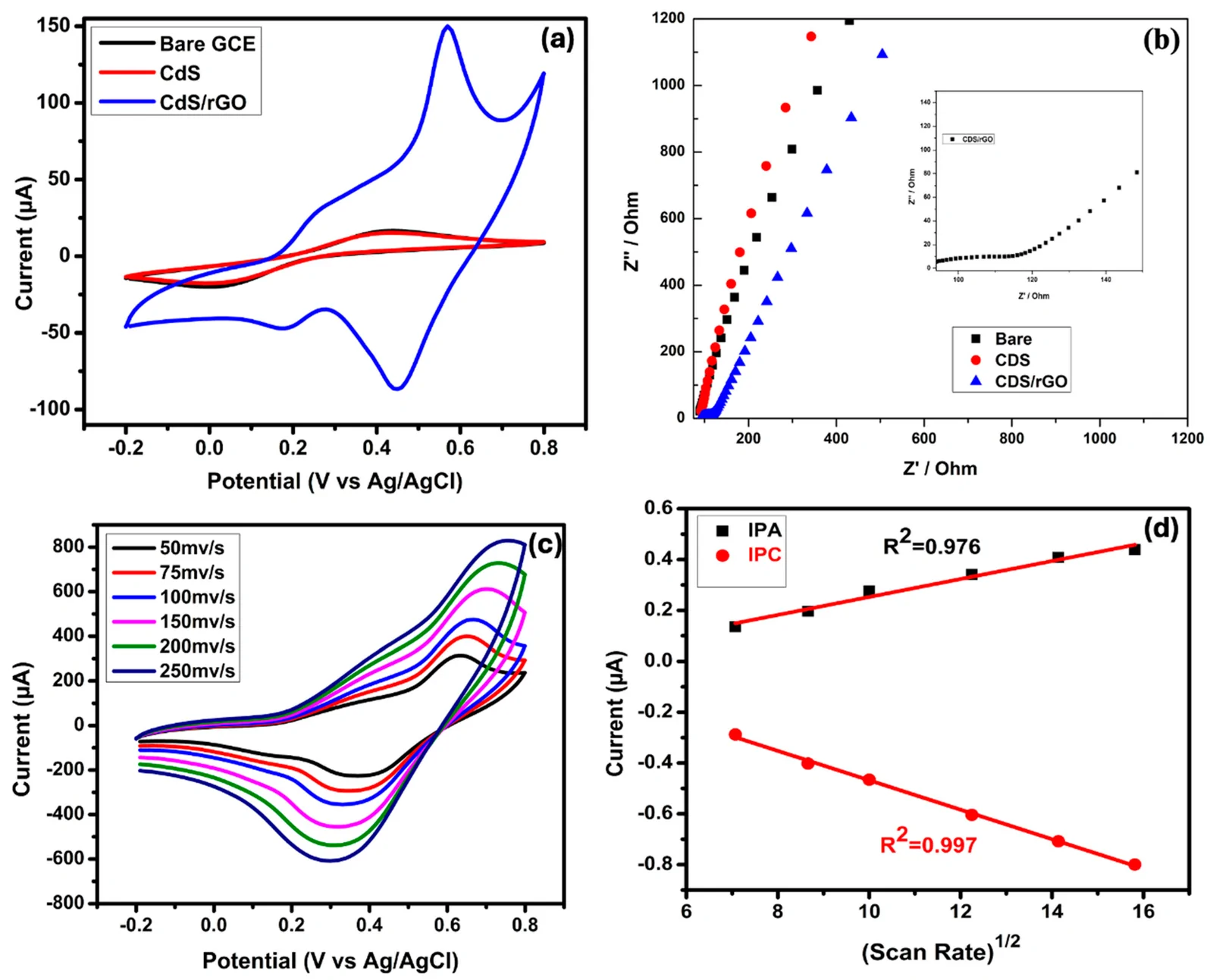
(**a**) CV curves and (**b**) Nyquist plots of the bare GCE, CdS-modified GCE, and CdS/rGO-modified GCE recorded in a 5 mM [Fe(CN)_6_]^3-/4-^ redox probe solution; the inset shows an enlarged Nyquist plot of the CdS/rGO-modified GCE. (**c**) CV responses of the CdS/rGO-modified GCE at different scan rates (50–250 mV s^−1^) and (**d**) the corresponding linear plots of anodic and cathodic peak currents versus v^1/2^.

The electrochemical properties of the bare GCE, CdS-modified GCE, and CdS/rGO-modified GCE were studied using CV and EIS in a 5 mM [Fe(CN)_6_]^3-/4-^ solution, as shown in Figure 3a–d. The CV response of the CdS-modified electrode exhibited poorly defined redox peaks compared with the bare GCE, which can be attributed to the relatively low electrical conductivity and possible aggregation of CdS nanoparticles, resulting in hindered electron-transfer kinetics and slightly less efficient electron transfer (Figure 3a). In contrast, the CdS/rGO-modified GCE exhibited well-defined redox peaks with significantly enhanced current responses and a smaller peak-to-peak separation (ΔEp = 120 mV) compared with the CdS-modified electrode (ΔEp = 420 mV). The electrochemically active surface area (ECSA) of both the bare GCE and the CdS/rGO-modified GCE was evaluated utilizing the Randles–Sevcik equation for a reversible redox process.

Ip = 2.69 × 10^5^ n^3/2^ A.D^1/2^ C.ν^1/2^

where

Ip = peak current for bare GCE and CdS/rGO/GCE (17.33 µA and 135.54 µA);

n = number of electron transferred (n = 1);

A = electroactive surface area (cm^2^);

D = diffusion coefficient of the [Fe(CN)_6_]^3−/4−^ (7.6 × 10^−6^ cm^2^ s^−1^);

C = concentration of the probe solution (5 mM or 5 × 10^−6^ mol /cm^3^);

ν = Scan rate (50 mV/s or 0.050 V/s).

Rearranging the equation:

A = Ip/2.69 × 10^5^ n^3/2^ D^1/2^ C.ν^1/2^

After substituting the values, the ECSA was determined to be 0.02009 cm^2^ for the bare GCE and 0.1635 cm^2^ for the CdS/rGO/GCE. The approximately 7.8-fold increase in ECSA following CdS/rGO modification confirms the substantially greater electrochemically accessible surface area of the composite-modified electrode. Thus, incorporation of highly conductive rGO increases the ECSA and facilitates charge transfer, thereby enhancing the electrocatalytic activity of the composite electrode [45]. Further insights into the electron-transfer characteristics were obtained using EIS measurements over a frequency range of 0.1 Hz to 10 kHz at a DC potential of 0.2 V vs. Ag/AgCl. The corresponding Nyquist plots (Figure 3b) consist of two characteristic regions: a high-frequency semicircle associated with the charge-transfer resistance (R_ct_) and a low-frequency linear region corresponding to the Warburg diffusion process (Z_W_) [46,47]. Notably, the CdS/rGO-modified electrode exhibited a substantially smaller semicircle and, consequently, a considerably lower R_ct_ compared with both the bare GCE and CdS-modified GCE. The spectra were fitted to the equivalent circuits: a Randles circuit, R_s_ + Q_dl_‖(R_ct_ + W), for the bare and CdS-modified GCE, and the same circuit with an additional film element (Q_f_‖R_f_) for the CdS/rGO-modified GCE, which accounts for the capacitive response of the porous rGO network. The resulting R_ct_ values are 18.1 kΩ, 21.5 kΩ, and 23.7 Ω for the bare GCE, CdS/GCE, and CdS/rGO/GCE, respectively, corresponding to a decrease of almost three orders of magnitude upon incorporation of rGO. The choice of equivalent circuits is discussed in detail in the Supplementary Information (Section S.2, Figures S3 and S4, Table S5), including a comparison with the Randles circuit fitted to the high-frequency region of the CdS/rGO/GCE spectrum. This reduced charge-transfer resistance indicates more efficient electron transfer between the electrode surface and the redox probe, demonstrating the beneficial role of rGO in enhancing the electrical conductivity and charge-transfer kinetics of the CdS/rGO composite. These EIS results are consistent with the smaller ΔE and higher peak currents observed in the CV measurements. The inset in Figure 3b provides an enlarged view of the Nyquist response of the CdS/rGO-modified electrode for greater clarity.

Cyclic voltammetry (CV) measurements were further performed at different scan rates ranging from 50 to 250 mV s^−1^ to investigate the electrochemical behavior and electron-transfer characteristics of the CdS/rGO-modified GCE, as shown in Figure 3c. Both the anodic (I_pa_) and cathodic (I_pc_) peak currents increased progressively with increasing scan rate, indicating enhanced mass transport of the redox species toward the electrode surface. Meanwhile, the peak-to-peak potential separation (ΔE) increased with increasing scan rate, suggesting a quasi-reversible electron-transfer process with increasing kinetic limitations at higher scan rates, consistent with the principles described by Laviron’s theory [48,49]. As shown in Figure 3d, the anodic and cathodic peak currents exhibited a linear relationship with the square root of the scan rate (v^1/2^). The corresponding linear regression equations were y = 35.351x − 100.88 for the anodic response and y = −57.949x + 111.46 for the cathodic response, with R^2^ values of 0.976 and 0.997, respectively. This linear dependence indicates that the electrochemical response of the CdS/rGO/GCE toward the [Fe(CN)_6_]^3-/4-^ redox probe is predominantly controlled by diffusion. The observed behavior confirms the favorable electrochemical characteristics of the CdS/rGO-modified electrode, including efficient charge transfer and electrochemical accessibility.

To achieve a low detection limit and good sensitivity toward heavy metal ions, several experimental parameters, including the supporting electrolyte, pH, deposition potential, and accumulation time, were systematically optimized to maximize the electrochemical response of the CdS/rGO-modified GCE, as shown in Figure S2a–d. In this study, Cu^2+^ ions were detected using differential pulse anodic stripping voltammetry (DPASV). Among these parameters, the choice of supporting electrolyte and pH is particularly important because they can influence the electrochemical potential window, metal-ion speciation, and hydrolysis behavior of heavy metal ions [50]. The effect of different supporting electrolytes on the DPASV response of the CdS/rGO-modified GCE toward Cu^2+^ was investigated using 0.1 M acetate, citrate, phosphate-buffered saline (PBS), and Tris-HCl solutions at pH 6, as shown in Figure S2a. The acetate buffer produced a well-defined Cu^2+^ stripping peak with a substantially higher current response than the other electrolytes. In contrast, weak current responses were observed in the Tris-HCl and PBS solutions. Therefore, acetate buffer was selected as the most suitable supporting electrolyte for subsequent experiments. The effect of pH on the Cu^2+^ stripping response was subsequently investigated in acetate buffer over the pH range of 3–7, as presented in Figure S2b. The anodic peak current increased progressively with increasing pH and reached a maximum at pH 6. This enhancement may be attributed to more favorable interactions between Cu^2+^ ions and the functional groups present on the CdS/rGO-modified electrode surface. However, a further increase in pH resulted in a decrease in the stripping current, which can be associated with the hydrolysis of Cu^2+^ and the possible formation of insoluble copper hydroxide species [51]. Accordingly, 0.1 M acetate buffer at pH 6 was selected as the optimum supporting electrolyte for subsequent electrochemical measurements.

The deposition potential is another critical parameter governing the stripping current of Cu^2+^ ions. To determine the optimum deposition potential, a potential range of 0 to −1.4 V was investigated while maintaining a constant deposition time of 120 s in 0.1 M acetate buffer (pH 6), as shown in Figure S2c. The stripping current increased progressively with increasing negative deposition potential and reached a maximum at −0.8 V, indicating efficient electrochemical reduction and accumulation of Cu^2+^ ions on the electrode surface. However, further shifting the deposition potential from −0.8 to −1.4 V resulted in a substantial decrease in the stripping current. This decrease can be attributed to the onset of hydrogen evolution at more negative potentials, which competes with Cu^2+^ deposition and consequently reduces the efficiency of analyte accumulation [52]. Therefore, −0.8 V was selected as the optimum deposition potential to achieve efficient Cu^2+^ accumulation while minimizing interference from hydrogen evolution. The effect of deposition time on Cu^2+^ accumulation was subsequently investigated over the range of 20–140 s, as shown in Figure S2d. The stripping peak current increased progressively with increasing deposition time and reached a maximum at 80 s, indicating enhanced accumulation of Cu^2+^ ions on the electrode surface. Beyond 80 s, no significant increase in peak current was observed, suggesting that the available active sites on the electrode surface had approached saturation, limiting further Cu^2+^ accumulation. Accordingly, a deposition time of 80 s was selected as the optimum condition for subsequent Cu^2+^ detection using the CdS/rGO-modified GCE.

### 2.4. Determination of Cu^2+^ Ions

**Figure 4:**
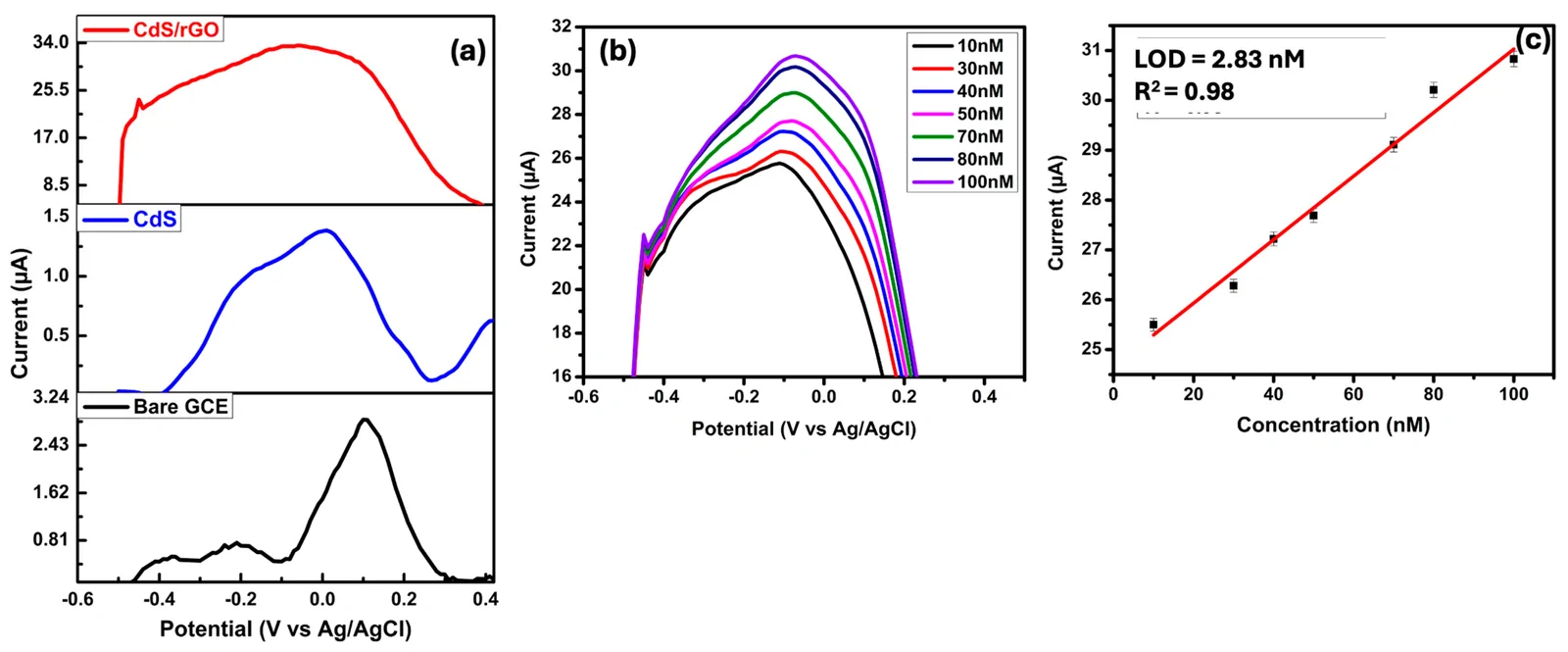
(**a**) DPASV responses of bare GCE, CdS-modified GCE, and CdS/rGO/GCE in 0.1 M acetate buffer (pH 6). (**b**) DPASV curves of the CdS/rGO-modified GCE at varying Cu^2+^ concentrations in 0.1 M acetate buffer (pH 6). (**c**) Corresponding linear calibration plot of peak oxidation current versus Cu^2+^ concentration.

The electrochemical responses of the prepared electrodes toward Cu^2+^ ions were evaluated using DPASV in 0.1 M acetate buffer (pH 6). As shown in Figure 4a, the bare GCE exhibited no appreciable stripping response toward 100 nM Cu^2+^, whereas the CdS-modified GCE showed only a weak stripping peak at approximately 0.01 V. In contrast, the CdS/rGO-modified GCE exhibited a sharp and well-defined stripping peak at approximately −0.1 V, accompanied by a substantially enhanced current response. The superior electrochemical performance of the CdS/rGO-modified electrode can be attributed to the synergistic effects of its structural and electronic properties. The highly conductive rGO framework provides efficient pathways for electron transport, while the CdS nanoparticles offer abundant electroactive sites that facilitate Cu^2+^ accumulation and subsequent electron-transfer reactions. The intimate integration of CdS with rGO therefore promotes efficient charge transfer and enhances the overall sensing response. Under the optimized experimental conditions, the CdS/rGO-based sensor was subsequently employed for the quantitative determination of Cu^2+^ ions over a range of concentrations. The corresponding DPASV responses and calibration curve are presented in Figure 4b and Figure 4c, respectively.

The oxidation peak current increased progressively with increasing Cu^2+^ concentration from 10 to 100 nM, demonstrating a clear concentration-dependent response. As illustrated in Figure 4c, the peak current response exhibited a well-defined concentration dependence toward Cu^2+^. The corresponding linear regression was expressed as (y = 0.063x + 24.65), with the slope of 0.063 µA nM^−1^ and an intercept of 24.65 µA. The regression analysis yielded an R^2^ value of 0.980, with 3% error bars obtained from three independent measurements. Based on the calibration response, the limit of detection (LOD), limit of quantification (LOQ), and sensitivity were determined as analytical performance parameters. The sensitivity was calculated as 0.901 µA nM^−1^ cm^−2^, whereas the LOD and LOQ were obtained using (3σ/S) and 10σ/S), respectively, while σ denotes the standard deviation of the blank response and (S) represents the calibration slope. The resulting LOD and LOQ values were 2.83 nM and 9.42 nM, respectively. Moreover, the obtained LOD is substantially lower than the reported regulatory limit for Cu^2+^ in drinking water, demonstrating the capability of the proposed sensor for trace-level Cu^2+^ detection. Moreover, the analytical performance of the CdS/rGO/GCE compares favorably with previously reported photoelectrochemical Cu^2+^ sensors, including ITO/CdS-rGO photoelectrodes [53]. A detailed comparison of the analytical performance of the proposed CdS/rGO/GCE sensor with other previously reported modified electrodes for Cu^2+^ detection is presented in Table 1. Overall, the results demonstrate that the proposed CdS/rGO/GCE offers competitive analytical performance, with a low detection limit and broad applicability for sensitive Cu^2+^ determination.

### 2.5. Reproducibility, Selectivity, and Stability

The reproducibility of the CdS/rGO-modified electrodes was evaluated by fabricating three independent electrodes under identical optimized conditions and measuring their responses toward 100 nM Cu^2+^ in 0.1 M acetate buffer (pH 6) (Figure S5). The calculated relative standard deviation (RSD) was 2.10%, demonstrating good agreement among the measurements and confirming the excellent reproducibility and consistency of the electrode fabrication process.

**Figure 5:**
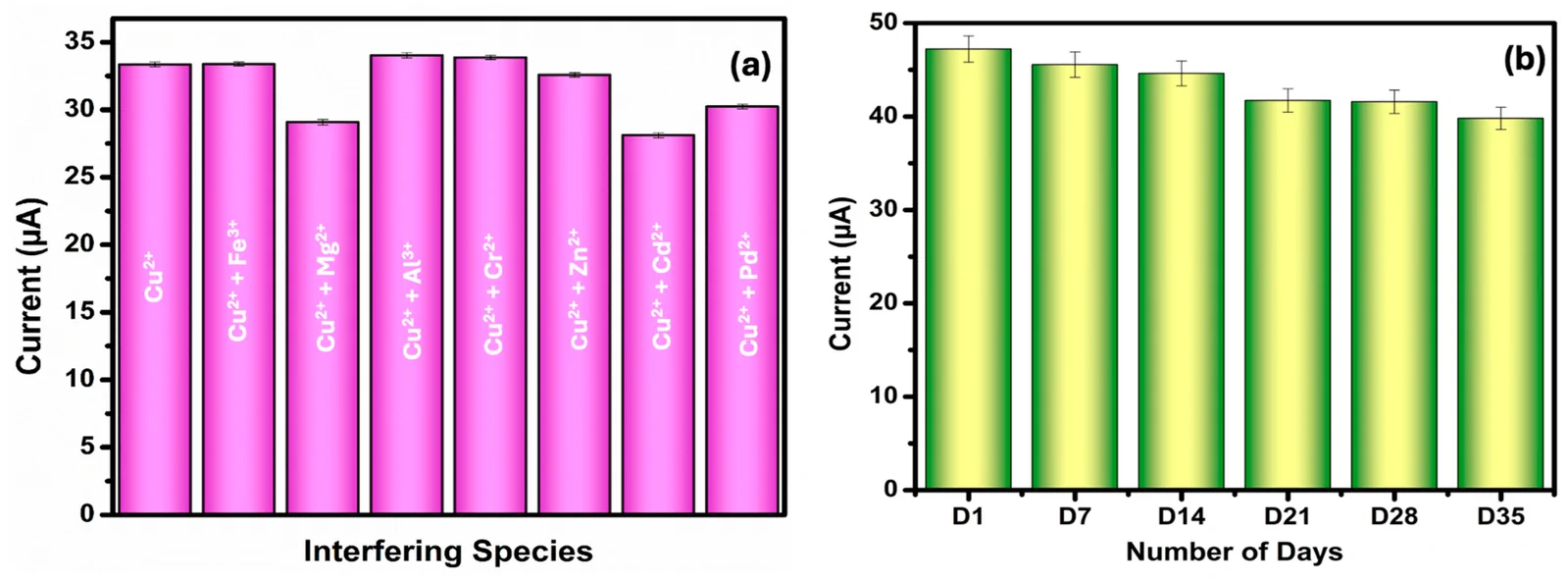
(**a**) Selectivity evaluation of the CdS/rGO/GCE toward 10 μM Cu^2+^ in the presence of 50 μM interfering ions (Mg^2+^, Fe^3+^, Zn^2+^, Al^3+^, Cr^3+^, Cd^2+^ and Pd^2+^). (**b**) Long-term stability of the sensor over 35 days using a single electrode, showing 84.27% retention of the initial response for Cu^2+^ sensing.

The analytical selectivity of the CdS/rGO sensor was evaluated in the presence of representative interfering metal ions, as shown in Figure 5a. The interference response of the CdS/rGO/GCE toward Cu^2+^ was quantitatively evaluated in the presence of 50 µM Mg^2+^, Fe^3+^, Zn^2+^, Al^3+^, Cr^3+^, Cd^2+^, and Pd^2+^ using 10 µM Cu^2+^ as the target analyte. Relative to the Cu^2+^ stripping current of 33.35 µA in the absence of interferents, the Cu^2+^ signal varied by −12.80%, +0.09%, −2.31%, +2.04%, +1.56%, −8.96%, and −5.94% in the presence of Mg^2+^, Fe^3+^, Zn^2+^, Al^3+^, Cr^3+^, Cd^2+^, and Pd^2+^, respectively. The limited variation in the Cu^2+^ stripping response for most investigated ions indicates that the CdS/rGO/GCE exhibits favorable tolerance toward these coexisting metal ions, with Mg^2+^ and Cd^2+^ producing the most pronounced signal attenuation.

The long-term stability of the developed electrochemical sensor was also investigated by monitoring its response toward 100 nM Cu^2+^ after storage at room temperature. As shown in Figure 5b, the sensor retained 84.27% of its initial current response after 35 days. This relatively small decrease in response demonstrates the good long-term stability of the CdS/rGO-based sensor and highlights its potential for practical Cu^2+^ sensing applications over extended periods.

### 2.6. Functional Group-Assisted Detection Mechanism of Cu^2+^

**Scheme 1:**
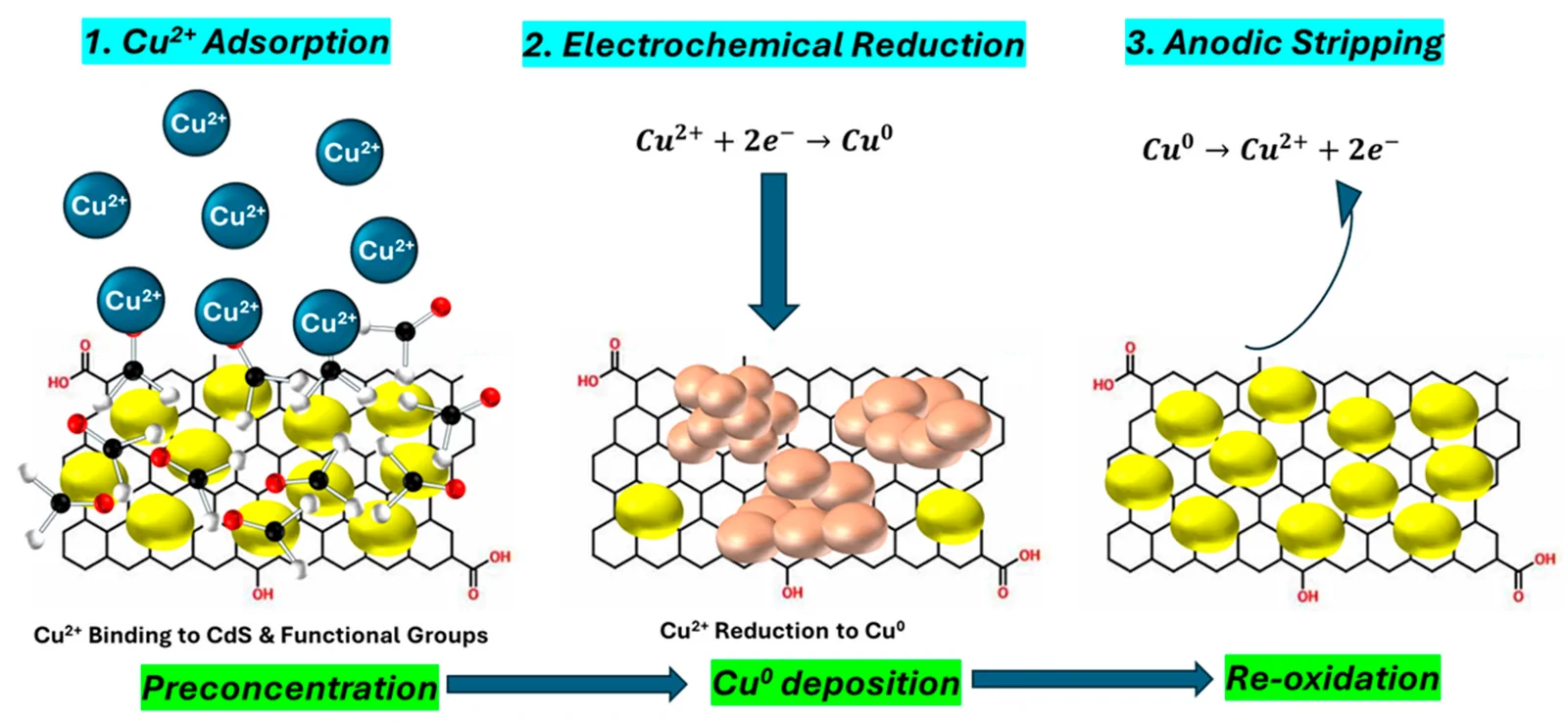
Mechanistic overview of Cu^2+^ detection via DPASV at the CdS/rGO electrode, including ion adsorption, electron transfer reduction, and anodic oxidation.

The Cu^2+^ detection mechanism at the CdS/rGO/GCE interface is proposed to involve Cu^2+^ accumulation at the modified electrode surface followed by electrochemical reduction and anodic stripping, as illustrated in Scheme 1. The XPS analysis confirms the presence of sulfur-containing species associated with CdS and oxygen-containing functional groups (-COOH, -OH, and C=O) associated with rGO. These surface functionalities may provide active sites for Cu^2+^ interactions and contribute to its accumulation during the preconcentration step.

During the application of the negative deposition potential, the accumulated Cu^2+^ is electrochemically reduced to metallic copper (Cu^0^) at the electrode surface, according to Cu^2+^ + 2e^−^ → Cu^0^. During the subsequent positive potential scan, the deposited Cu^0^ is oxidized back to Cu^2+^, accompanied by the release of electrons (Cu^0^ → Cu^2+^ + 2e^−^), generating the anodic stripping peak used for quantitative determination. The enhanced electrochemical response of the CdS/rGO/GCE may be attributed to its increased electrochemically accessible surface area, conductive rGO framework, and favorable surface characteristics, which collectively facilitate Cu^2+^ accumulation and electron transfer.

### 2.7. Application to Real Sample Testing

To assess the practical applicability of the proposed CdS/rGO-based electrochemical sensor, Cu^2+^ concentrations of 20, 40, and 60 nM were investigated by DPASV. Tap water, mineral water, and a carbonated beverage were selected as real sample matrices because of their distinct chemical compositions and potential trace levels of Cu^2+^. The analysis was intended to determine whether the modified electrode could generate a reliable and distinguishable stripping response for trace Cu^2+^ in actual sample matrices, rather than only in standard solutions. Prior to analysis, each sample was diluted tenfold with 0.1 M acetate buffer (pH 6) at a 1:9 sample-to-buffer ratio and subsequently spiked with known concentrations of Cu^2+^. The Cu^2+^ concentrations in the prepared samples were determined using DPASV, and the corresponding recovery values are summarized in Table 2.

The obtained average recoveries ranged from 71.20% to 130.20%, demonstrating satisfactory accuracy and reliability of the developed sensor in complex sample matrices. Furthermore, successful detection in these matrices demonstrates the capability of the CdS/rGO electrode for trace-Cu^2+^ analysis and supports its potential application in broader real-sample trace-metal monitoring.

## 3. Conclusions

In this study, a highly sensitive, efficient, and reliable CdS/rGO-modified GCE was successfully fabricated for the electrochemical detection of Cu^2+^ ions using the DPASV technique. The integration of CdS nanoparticles with the conductive rGO matrix enhanced the electrical conductivity of the sensing platform while providing abundant electroactive sites for Cu^2+^ accumulation, thereby facilitating efficient electron transfer and improving the overall electrochemical response. Under optimized experimental conditions, the CdS/rGO/GCE sensor exhibited a linear response over the concentration range of 10–100 nM, with a low limit of detection (LOD) of 2.83 nM for Cu^2+^ ions. The sensor also demonstrated good selectivity toward Cu^2+^ in the presence of potentially interfering metal ions, together with good reproducibility (RSD = 2.10%) and satisfactory long-term stability. Furthermore, the proposed sensor showed reliable performance in real water samples and complex matrices, achieving satisfactory recovery values. Overall, the results demonstrate that the CdS/rGO/GCE is a promising sensing platform for the sensitive, selective, and reliable determination of Cu^2+^ ions, with potential applications in environmental monitoring and analytical detection.

## Figures and Tables

**Table 1:** Comparison of the CdS/rGO/GCE with other previously reported electrode materials for the detection of Cu^2+^.

Electrodes	Methods	Linear Range	LOD	Reference
PCN-224/rGO@Au/GCE	DPASV	5–150 μM	1.5 μM	[54]
RGO/Bi/GCE	DPASV	0.3–1.5 μM	0.41 μM	[55]
CS/GO/GCE-IIP/GCE	DPASV	0.5–100 μM	0.15 μM	[56]
PbS/GNS/GCE	CV	1 × 10^−4^ to 1 × 10^−8^ M	1 × 10^−8^ M	[30]
MoS_2_/SPCE	DPV	0.005~5 mM	5.43 µM	[57]
Fe_3_O_4_-GSH/CHI/GCE	DPV	10~200 nM	4.83 nM	[58]
RGO/NiWO_4_	DPASV	0.2 to 0.7 μM	2.3 × 10^−10^ M	[59]
ITO/CdS-rGO	Photoelectrochemical	0.5–120 µM	0.016 µM	[53]
PGA/GO/GCE	SWASV	0.25–5.5 µM	0.02 µM	[60]
ZIF-67/GO/Na/GCE	SWASV	0.01–1 7.5 μM	0.005 μM	[61]
Kryptofix 21-capped GNPs/RGO/GCE	DPASV	0.5–75 μM	0.1 μM	[62]
CdS/rGO/GCE	DPASV	10–100 nM	2.83 nM	Present work

**Table 2:** Quantification of Cu (II) in environmental water samples and carbonated beverages using the proposed method.

Samples	Added (nM)	Found (nM)	Recovery (%)	%RSD (*n* = 3)
Tap Water	0	0.99	…	…
	20	14.25	71.25	3.16
	40	42.69	106.73	3.72
	60	56.27	93.78	5.78
Carbonated drink	0	2.64	…	…
	20	15.70	78.51	3.82
	40	41.81	104.54	3.61
	60	61.12	101.87	2.90
Mineral Water	0	0	…	…
	20	26.04	130.20	2.46
	40	37.01	92.52	3.94
	60	60.61	101.02	2.99

## Data Availability

The original contributions presented in this study are included in the article/Supplementary Materials. Further inquiries can be directed to the corresponding author.

## Supplementary Materials

The following supporting information can be downloaded at https://www.mdpi.com/article/10.3390/s26216729/s1. Experimental Section, Scheme S1: Schematic overview of the preparation of CdS/rGO nanocomposites [33] and their electrochemical sensing mechanism for the determination of Cu^2+^ ions. Figure S1: Deconvoluted high-resolution XPS profiles of (a) overall survey spectrum of CdS/rGO, (b) Cd 3d, (c) S 2p, (d) C 1s, and (e) O 1s [63]. Figure S2: (a–d) Optimization of the supporting electrolyte, pH, deposition potential, and deposition time, respectively, for DPASV-based detection of 50 μM Cu^2+^. Figure S3: Equivalent-circuit fits the EIS spectra of the bare GCE (left), CdS/GCE (center), and CdS/rGO/GCE (right) in 5 mM [Fe(CN)_6_]^3−/4−^ at 0.2 V vs. Ag/AgCl. Figure S4: CdS/rGO/GCE: parameters of the Randles circuit fitted from a lowest frequency f_min_ up to 10 kHz (symbols, ± 1σ), compared with the Randles + film circuit fitted to the full spectrum (line, ± 1σ band). Figure S5: Raw DPASV for reproducibility of CdS/rGO/GCE for Cu^2+^ sensing. Table S1: Parameters of the Cd 3d doublets. Table S2: Parameters of the S 2p doublets. Table S3: Parameters of the C 1s components. Table S4: Parameters of the O 1s components. Table S5: Fitted equivalent-circuit parameters (± 1σ) and rms deviations of the EIS spectra.
