# Supplementary material for: CdS/rGO Nanocomposite for Sensitive Electrochemical Detection of Copper Ions
Source: Sensors (Basel). 2026 Oct 24;26(21):6729. doi: 10.3390/s26216729 (PMC13765858; doi:10.3390/s26216729)
Supplement: Supplementary file 1 [file sensors-26-06729-s001.zip › sensors-4559237-supplementary.pdf]

Supporting Information

CdS/rGO Nanocomposite for Sensitive Electrochemical Detection of Copper Ions

Muhammad Sajid Shafiq[a], Fabian O. Romero-Soto[a], Martín F. Jiménez-Moreno[a], Amritha kadathamana [c], Masoud Madadelahi[a], Marc J. Madou [a,b], Josué Amilcar Aguilar Martínez [c,d] and Rudra Kumar[a] *

[a] School of Engineering and Sciences, Tecnologico de Monterrey, Av. Eugenio Garza Sada 2501 Sur, Monterrey 64849, NL, Mexico

[b] Department of Mechanical and Aerospace Engineering, University of California, Irvine, CA 92697, USA

[c] Centro de Investigación e Innovación en Ingeniería Aeronáutica (CIIIA), Facultad de Ingeniería Mecánica y Eléctrica, Universidad Autónoma de Nuevo León, Carretera a Salinas Victoria km. 2.3, Apodaca 66600, Nuevo León, México;

[d] Facultad de Ingeniería Mecánica y Eléctrica, Universidad Autónoma de Nuevo León, Av. Universidad, Cd. Universitaria, San Nicolás de los Garza, Nuevo León, México, 66455.

S.1 Experimental Section

S.1.1 Materials

All chemicals and reagents were obtained commercially from Sigma-Aldrich and employed directly without any additional purification. The chemicals included magnesium chloride, iron chloride, zinc acetylacetonate hydrate ($Zn(C_5H_7O_2)_2 \cdot xH_2O$, 99.995%), ammonium hydroxide (NH_4OH), thiourea (CH_4N_2S, ≥ 99.0%), aluminum nitrate nonahydrate ($Al(NO_3)_3 \cdot 9H_2O$, ≥ 98%), cadmium acetate dihydrate ($Cd(CH_3COO)_2 \cdot 2H_2O$, ≥ 99.0%), chromium nitrate nonahydrate ($Cr(NO_3)_3 \cdot 9H_2O$, ≥ 99.99%), graphite flakes, citric acid ($C_6H_8O_7$, ≥ 99.5%), acetic acid, and sodium acetate anhydrous (CH_3COONa, ≥ 99.0%).

S.1.2. Chemical Synthesis of Graphene Oxide (GO)

Graphene oxide (GO) was synthesized from graphite flakes using a modified Hummers method, as previously reported in the literature [1]. Briefly, 1 g of graphite flakes was mixed with 1 g of sodium nitrate, followed by the careful addition of 46 mL of concentrated sulfuric acid (H_2SO_4) under continuous stirring in an ice bath at approximately 10 °C for 10 min. Subsequently, 6 g of potassium permanganate ($KMnO_4$) was added gradually to the reaction mixture under constant stirring while maintaining the temperature below 20 °C to ensure controlled oxidation. After thorough mixing, the reaction mixture was transferred to a water bath and maintained at 35 ± 5 °C under continuous stirring for approximately 5 h, resulting in the formation of a thick, viscous paste. Subsequently, 80 mL of deionized water (DIW) was slowly added to the mixture under continuous stirring for 30 min, during which the temperature increased to approximately 95 °C. The resulting suspension was further diluted with 200 mL of DI water, followed by the careful addition of 6 mL of 30% hydrogen peroxide (H_2O_2) to terminate the oxidation reaction. A distinct color change from dark brown to yellow was observed, indicating the formation of GO. The resulting product was filtered and thoroughly washed several times with 30% hydrochloric acid (HCl) and DI water to remove residual acidic and inorganic impurities. The suspension was then subjected to low-speed centrifugation (≈1000 rpm) for 2-3 cycles to remove larger unreacted particles. The collected supernatant was subsequently centrifuged at approximately 8000 rpm for 15 min to remove remaining water-soluble by-products. Finally, the purified material was dried in an oven at 60 °C overnight to obtain GO as a dry solid.

S.1.3. Synthesis of Cadmium Sulfide (CdS) Nanoparticles

CdS nanoparticles were synthesized using a one-pot hydrothermal method. Briefly, 10 mmol of thiourea and 1 mmol of cadmium acetate were dissolved in 70 mL of deionized water (DIW) under continuous stirring. Subsequently, 1 M ammonium hydroxide (NH_4OH) solution was added dropwise to adjust the pH of the reaction mixture to approximately 7.0. The resulting solution was continuously stirred for 2 h to ensure complete dissolution and homogeneous mixing of the precursors. The mixture was then transferred into a Teflon-lined stainless-steel hydrothermal reactor at 150 °C for 15 h. The resulting product was collected by centrifugation and thoroughly washed several times with ethanol and DIW to remove remaining impurities and unreacted species. The purified product was subsequently dried to obtain CdS nanoparticles.

S.1.4. Synthesis of CdS/rGO Nanocomposites

CdS/rGO nanocomposites were synthesized via a hydrothermal method. Briefly, 1 mmol of cadmium acetate and 7 mmol of thiourea were dissolved in 70 mL of deionized water (DIW) under continuous stirring. The pH of the resulting solution was adjusted to approximately 7.0 using 1 M ammonium hydroxide (NH_4OH). Subsequently, 50 mg of GO was added to the precursor solution and thoroughly dispersed by stirring for 2 h, followed by ultrasonication for 30 min to ensure uniform distribution of GO. The resulting homogeneous suspension was then transferred into a 70 mL Teflon-lined hydrothermal reactor and maintained at 150 °C for 15 h. After naturally cooling to room temperature, the resulting product was collected by centrifugation at 8000 rpm for 10 min and then washed several times with ethanol and DIW to remove residual impurities and unreacted species. Finally, the purified CdS/rGO nanocomposite was dried at 70 °C for 4 h to obtain the desired product. **Scheme 1** illustrates the synthesis route of the CdS/rGO nanocomposite and its proposed role in electrochemical sensing.

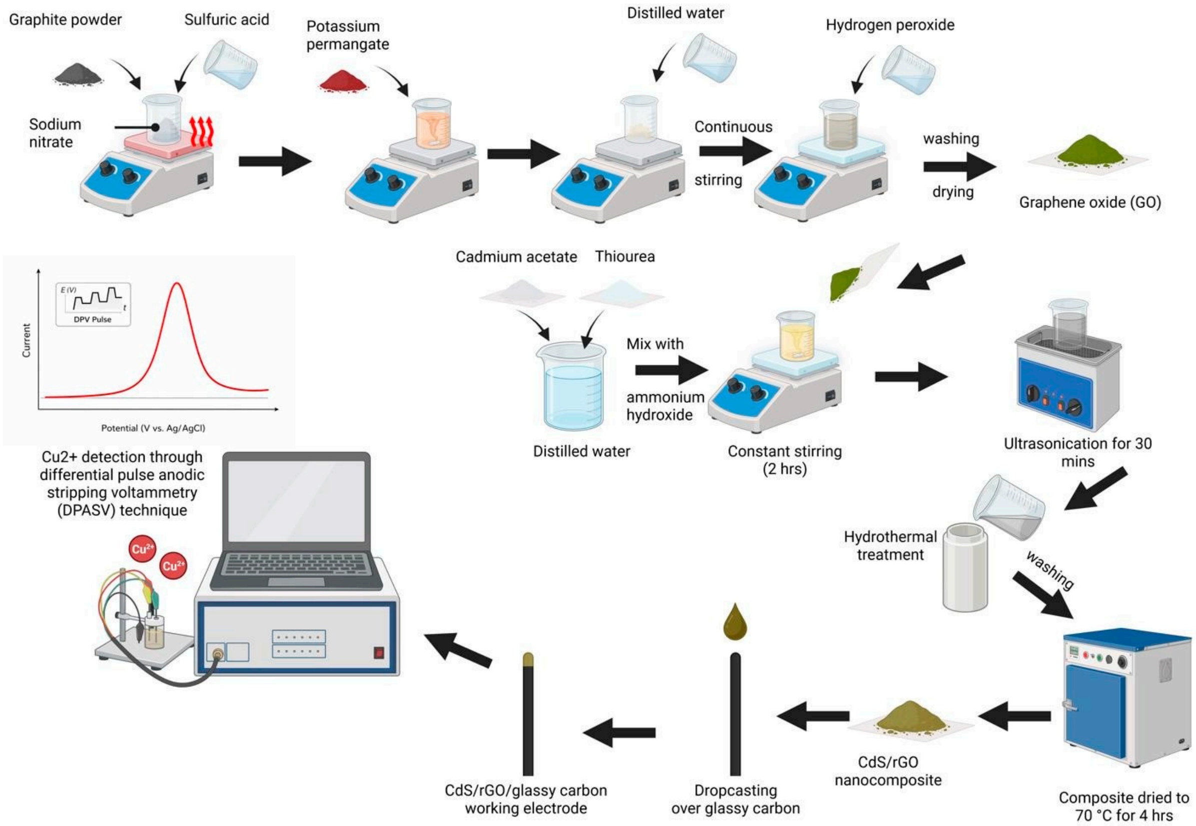

Scheme 1. Schematic overview of the preparation of CdS/rGO nanocomposites and their electrochemical sensing mechanism for the determination of Cu^{2+} ions.

S.1.5. Fabrication of the CdS/rGO/GCE

To prepare the electrode-modifying suspension, 5 mg mL^{-1} of the CdS/rGO nanocomposite was dispersed in 2 mL of deionized water (DIW) and ultrasonicated for 30 min to obtain a homogeneous suspension. Prior to modification, the glassy carbon electrode (GCE) surface was polished with alumina to obtain a smooth and clean surface, followed by thorough rinsing with DIW. Subsequently, 10 μL of the CdS/rGO

suspension was drop-cast onto the polished GCE surface and allowed to dry at room temperature. The resulting CdS/rGO-modified GCE (CdS/rGO/GCE) was used as the working electrode for subsequent electrochemical sensing experiments.

S.1.6. Characterization of Synthesized Materials

The phase structure of the prepared materials was characterized by X-Ray Diffraction (XRD) on a D2 Phaser diffractometer (Bruker Corporation, MA, USA) utilizing CuKα radiation (λ = 0.154 nm). Data acquisition was performed across a 2θ range of 10° to 90°. The morphological features were examined using FESEM (Gemini 3560, Germany). Elemental composition and distribution were inspected by Energy-Dispersive Xray spectroscopy (EDX) with a detector integrated into the FESEM system. EDX analysis and elemental mapping were carried out under an accelerating voltage of 15 kV, maintaining a working distance of 10 mm. Fourier transform infrared (FTIR) was employed to identify the characteristic functional groups. Spectra were acquired in the 4000-400 cm^{-1} range using a Frontier FTIR spectrometer equipped with a universal ATR sampling accessory (PerkinElmer, MA, USA). Further evaluation of surface composition and chemical states was carried out by X-ray photoelectron spectroscopy (Thermo-VG Scientific Co., USA). To probe the structural properties and defect density, Raman spectroscopy was employed using a confocal Xplora Macro Raman spectrometer (Horiba, Kyoto, Japan). The system was equipped with a 532 nm solidstate laser along with a CCD detector. Raman spectra were collected over a range of 500-4000 cm^{-1}.

S.1.7. Electrochemical Characterization

Electrochemical experiments were conducted with a CHI 760E potentiostat (Chenhua Instruments, Shanghai, China) employing a standard three-electrode cell. The electrochemical cell consisted of a GCE serving as the working electrode, a Pt wire functioning as the auxiliary electrode, and an Ag/AgCl electrode used as the reference electrode. Cyclic voltammetry (CV) was initially employed to investigate the electrontransfer kinetics of the fabricated electrodes using 5 mM $[Fe(CN)_6]^{3-/4-}$ as the redox probe. CV measurements were recorded over a potential window of -0.2 to 0.5 V at scan rates ranging from 50 to 250 mV s^{-1}. Electrochemical impedance spectroscopy (EIS) measurements were subsequently carried out in the same redox probe solution at a fixed DC potential of 0.2 V vs. Ag/AgCl, over a frequency range of 10 kHz to 0.1 Hz (50 logarithmically spaced frequencies) with an AC amplitude of 10 mV. The spectra were analyzed by equivalent-circuit fitting, as described in Section S.2. Differential pulse anodic stripping voltammetry (DPASV) was used under optimized experimental conditions for the detection of Cu^{2+} ions and evaluation of the analytical performance of the developed electrode. The DPASV measurements were performed using a conditioning potential of −1.1 V for 5 s, followed by a deposition potential of −0.8 V for 80 s. An equilibration time of 5 s was applied prior to the differential pulse scan. The potential was then scanned from −0.5 to +0.8 V, with a potential step of 10 mV, pulse amplitude of 100 mV, and pulse width of 0.02 s.

S.1.8. Real Sample Collection

To evaluate the reliability and practical applicability of the developed sensing platform, Cu^{2+} ions were determined in real samples, including tap water and carbonated beverages. Commercial mineral water and carbonated beverages were purchased from a local grocery store in Monterrey, Mexico, while tap water samples were collected directly from the laboratory. The collected samples were analyzed without further treatment unless otherwise specified.

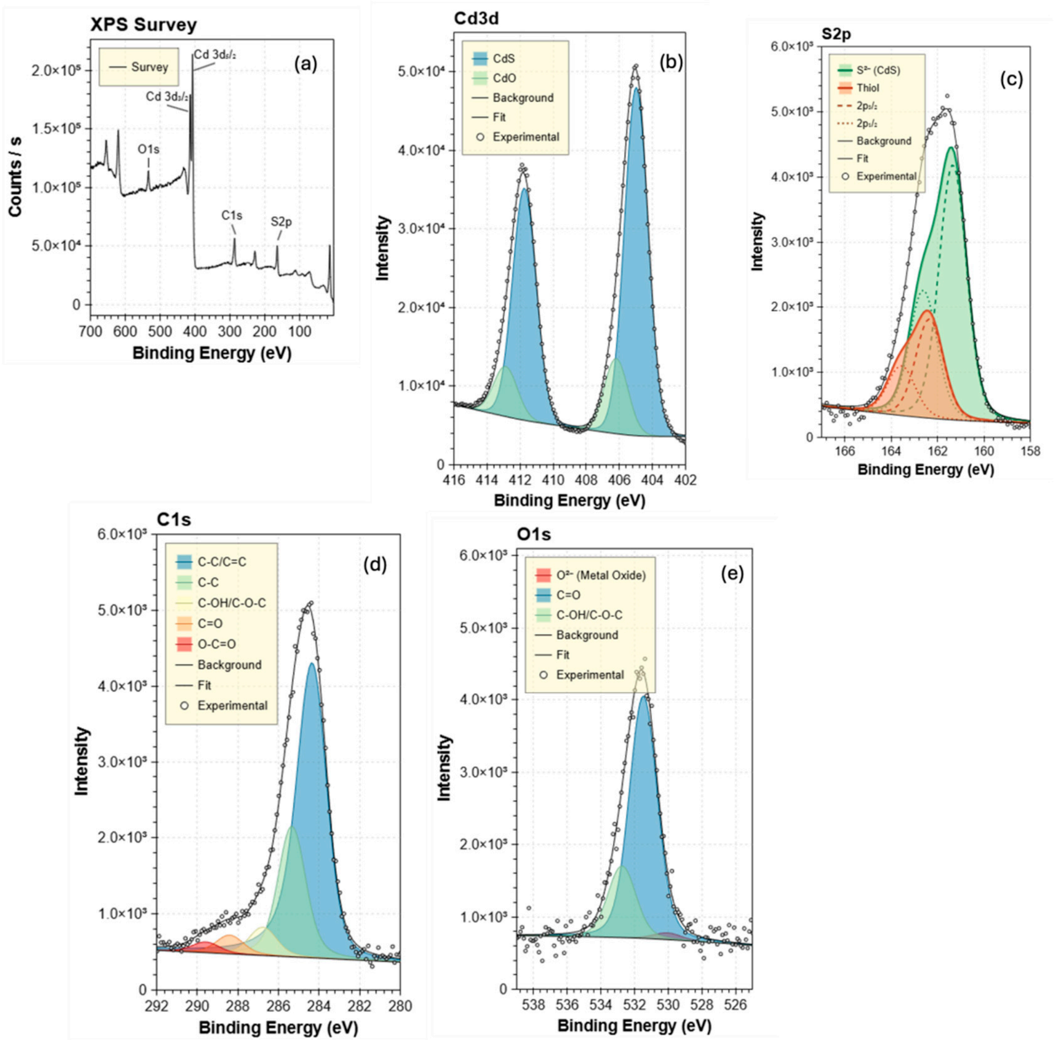

Fig. S1. Deconvoluted high-resolution XPS profiles of (a) overall survey spectrum of CdS/rGO, (b) Cd 3d, (c) S 2p, (d) C 1s, and (e) O 1s. In (c), each labelled component is an S 2p doublet; dashed and dotted lines show its $2p_{3/2}$ and $2p_{1/2}$ lines. The fit parameters of (b)–(e) are listed in Tables S1–S4.

Fitting of the High-Resolution XPS Spectra

The high-resolution Cd 3d, S 2p, C 1s and O 1s spectra (Fig. S1b–e) were fitted with AAnalyzer (Al Kα, with hν = 1486.7 eV). Binding energies were charge-referenced to the maximum of the C 1s spectrum at 284.6 eV. The background was modelled as a linear function plus an active two-parameter Tougaard background, refined simultaneously with the peaks, for Cd 3d, S 2p and O 1s, and as a linear function for C 1s. All components were described by Voigt profiles, except the sp^2 C 1s component, for which an asymmetric line shape (double Lorentzian convoluted with a Gaussian) was used to account for the asymmetric tail characteristic of graphitic carbon [2]. Within each region, all components share the same Gaussian and Lorentzian widths. The Cd 3d and S 2p components were fitted as spin–orbit doublets with the area ratios fixed at their theoretical values ($3d_{3/2}$:$3d_{5/2}$ = 2:3 and $2p_{1/2}$:$2p_{3/2}$ = 1:2), so that each label in Fig. S1b and Fig. S1c denotes a complete doublet. The fitted parameters of all components are listed in Tables S1–S4: binding energies (BEs) and areas of both spin–orbit lines and their separation (ΔSO) for the doublets, and the position of the maximum, full width at half maximum (FWHM) and area for the singlets. Cd 3d*:* Two doublets are resolved. The main doublet (Cd $3d_{5/2}$ at 404.98 eV, Cd $3d_{3/2}$ at 411.74 eV, ΔSO = 6.77 eV; 82% of the Cd 3d area) is assigned to Cd^{2+} in CdS. A second doublet with its $3d_{5/2}$ line at 406.19 eV (18%) is assigned to oxidized Cd (Cd–O species, labelled "CdO" in Fig. S1b), consistent with the metal–oxygen component of the O 1s spectrum and the oxidized-sulfur doublet of the S 2p spectrum. The label denotes oxidized Cd in general; CdO itself is usually reported at a binding energy equal to or lower than that of CdS, whereas this doublet lies 1.2 eV higher, closer to the values reported for $Cd(OH)_2$ and $CdSO_4$. Together with the sulfate-like S 2p doublet, this suggests that the oxidized Cd is present mainly as surface hydroxide or sulfate species.

Table S1. Parameters of the Cd 3d doublets (Fig. S1b). Areas in counts·eV; fractions refer to the total area of the region.

Component	$3d_{5/2}$ BE / eV	$3d_{3/2}$ BE / eV	ΔSO / eV	FWHM / eV	$3d_{5/2}$ area	$3d_{3/2}$ area	Fraction / %
CdS	404.98	411.74	6.77	1.68	80916	53890	82.3
CdO	406.19	412.88	6.68	1.68	17443	11617	17.7

S 2p. Three doublets were identified. The components labelled "S^{2-} (CdS)" and "Thiol" in Fig. S1c denote complete doublets; their individual $2p_{3/2}$ and $2p_{1/2}$ lines are drawn as dashed and dotted lines, respectively. The main doublet (S $2p_{3/2}$ at 161.34 eV, S $2p_{1/2}$ at 162.62 eV; 67%) is assigned to lattice sulfide in CdS. A second doublet (162.35/163.56 eV; 26%) is attributed to thiol-type C–S sulfur from residual thiourea-derived species, and a third, minor doublet (168.65/169.86 eV; 7%), outside the shown energy window of Fig. S1c, is attributed to oxidized sulfur. The feature near 162 eV is therefore the superposition of the sulfide $2p_{1/2}$ line and the thiol $2p_{3/2}$ line. The fitted spin–orbit splittings (1.21–1.27 eV) are comparable to the tabulated S 2p value (≈1.18 eV). A model with a single sulfide doublet cannot reproduce the high-binding-energy shoulder: it requires an unphysically broad Gaussian width (1.64 eV) and gives a higher reduced

χ^2 (4.83 vs. 4.33). The additional doublet significantly improves the fit (F-test, F = 17 for two additional parameters, $p \approx 10^{-7}$).

Table S2. Parameters of the S 2p doublets (Fig. S1c). Areas in counts·eV; fractions refer to the total area of the region.

Component	$2p_{3/2}$ BE / eV	$2p_{1/2}$ BE / eV	ΔSO / eV	FWHM / eV	$2p_{3/2}$ area	$2p_{1/2}$ area	Fraction / %
S^{2-} (CdS)	161.34	162.62	1.27	1.41	6350	3175	67.2
Thiol (C–S)	162.35	163.56	1.21	1.41	2469	1235	26.1
Oxidized S	168.65	169.86	1.21	1.41	627	313	6.6

C 1s. Five components are resolved: sp^2 C=C at 284.35 eV (64% of the C 1s area), sp^3 C–C at 285.34 eV (26%), and the oxygen-containing groups C–OH/C–O–C (286.77 eV), C=O (288.40 eV) and O–C=O (289.58 eV), which together account for 11%. The sp^2 component is asymmetric and therefore broader (FWHM 1.76 eV) than the symmetric components (1.61 eV).

Table S3. Parameters of the C 1s components (Fig. S1d). Areas in counts·eV; fractions refer to the total area of the region.

Component	BE / eV	FWHM / eV	Area	Fraction / %
C=C (sp^2)	284.35	1.76	8508	63.7
C–C (sp^3)	285.34	1.61	3430	25.7
C–OH/C–O–C	286.77	1.61	718	5.4
C=O	288.40	1.61	456	3.4
O–C=O	289.58	1.61	250	1.9

O 1s. Three components are resolved: metal–oxygen (O^{2-}) at 530.04 eV (2% of the O 1s area), C=O at 531.48 eV (76%) and C–OH/C–O–C at 532.75 eV (22%).

Table S4. Parameters of the O 1s components (Fig. S1e). Areas in counts·eV; fractions refer to the total area of the region.

Component	BE / eV	FWHM / eV	Area	Fraction / %
O^{2-} (metal oxide)	530.04	1.96	214	2.1
C=O	531.48	1.97	7644	75.7
C–OH/C–O–C	532.75	1.97	2238	22.2

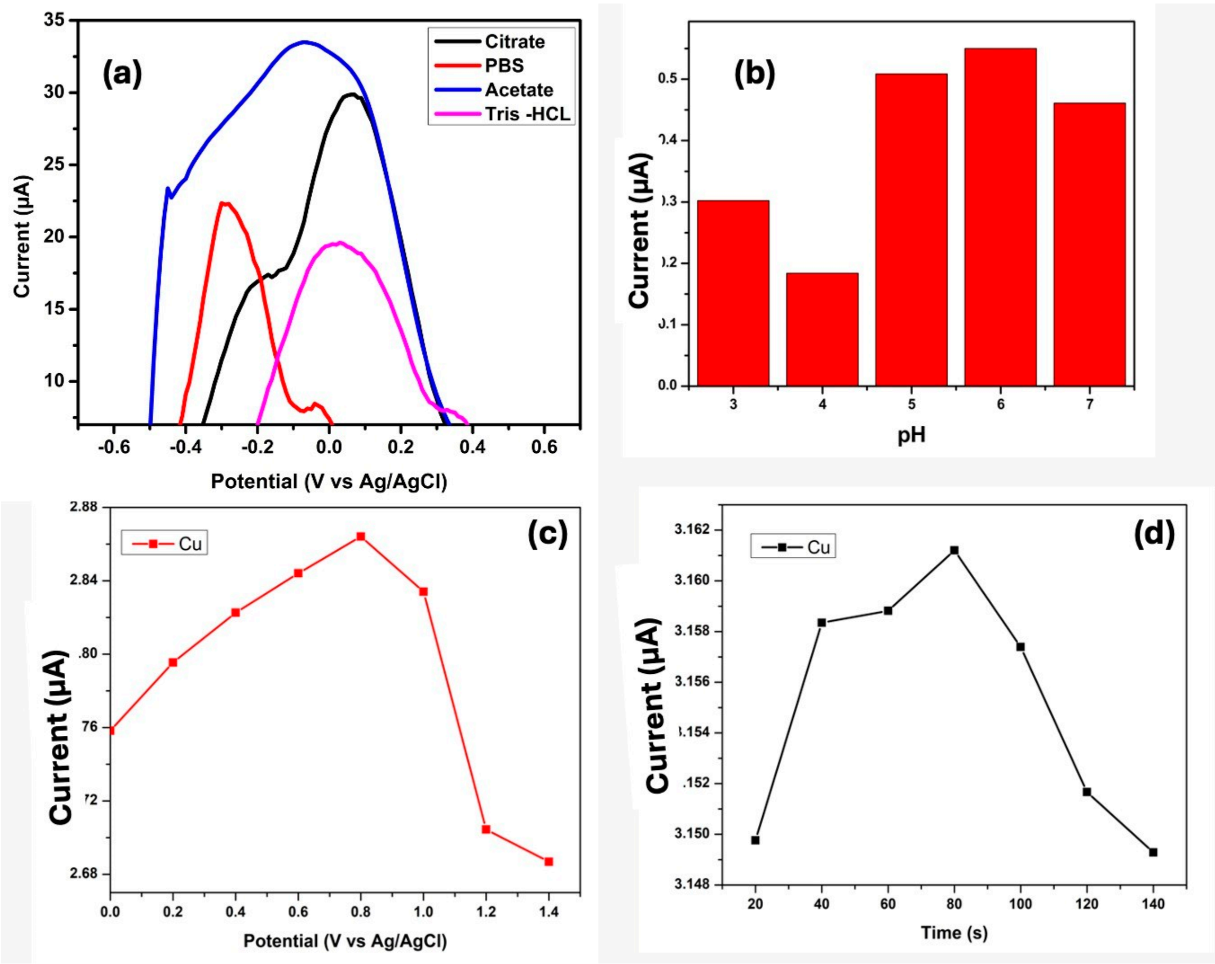

Fig. S2. (a-d) Optimization of the supporting electrolyte, pH, deposition potential, and deposition time, respectively, for DPASV-based detection of 50 μM Cu^{2+}.

S.2 Equivalent-Circuit Analysis of the EIS Spectra

The EIS spectra of the bare GCE, CdS/GCE, and CdS/rGO/GCE (Section S.1.7) were fitted to equivalent circuits by complex non-linear least squares with modulus weighting over the full frequency range (10 kHz–0.1 Hz), using a multi-start trust-region algorithm implemented in Python (SciPy). Constant-phase elements, $Z_{CPE} = 1/[Q(j\omega)^n]$, were used instead of ideal capacitors to account for the depressed semicircles typical of rough and porous electrodes. Parameter uncertainties (± 1σ) were obtained from the covariance matrix of the fit. The fits, residuals, and fitted parameters are shown in Fig. S4 and Table S5.

For the Bare GCE and CdS/GCE cases, both spectra are described by the Randles circuit, $R_s + Q_{dl} \parallel (R_{ct} + W)$, where R_s is the solution resistance, Q_{dl} the double-layer constant-phase element, R_{ct} the charge-transfer resistance, and W a semi-infinite Warburg element (rms deviation 1.2% and 1.4%, respectively). Adding a film element to these circuits did not yield well-determined parameters, as the exponents of the two

constant-phase elements and R_{ct} were strongly correlated ($|\varrho|$ = 0.97–0.99, from the covariance matrix of the fit) and R_f was determined only to within ±28–29%; the Randles circuit was therefore retained.

For CdS/rGO/GCE, the Randles circuit cannot describe the full spectrum of this electrode as the fit converges to an unphysical solution (R_{ct} at its upper bound, 5% rms deviation). Note that the chargetransfer semicircle is small (Z′ ≈ 91–115 Ω) and confined to high frequencies, where the phase angle remains at ≈ 5° from 2.4 kHz down to ≈ 230 Hz. Below ~100–200 Hz, the phase rises steeply (to ≈ 65° at 0.1 Hz), reflecting a large capacitive contribution that is not part of the Randles circuit. This contribution is attributed to the porous rGO network and is therefore described by an additional film element ($Q_f \| R_f$) in series with the Randles circuit. This extended circuit fits the full spectrum with a 0.3% rms deviation and residuals below 1% over the whole frequency range (Fig. S4). The film capacitance ($Q_f \approx 6.5 \times 10^{-4}$ S s^n, n_f = 0.87) is about 20 times larger than Q_{dl}, consistent with a high-surface-area porous layer.

The film element ($Q_f \| R_f$) accounts for the low-frequency response only and leaves the parameters unchanged. We demonstrate this by fitting the conventional Randles circuit to the high-frequency region alone (f ≥ 100 Hz), where the film contribution is negligible, yielding R_s = 90.1 Ω, R_{ct} = 22.9 Ω, $Q_{dl} = 3.12 \times 10^{-5}$ S s^n, and n_{dl} = 0.756, which is in agreement within 3.3% with the full spectrum fitted Randles + film circuit (respectively, 90.6 Ω, 23.7 Ω, 3.18×10^{-5} S s^n, and 0.764; consult Table S5 for better contrast). The Randles parameters vary with the lower frequency bound of the fit but remain in agreement within ~20% of the Randles + film values (R_{ct} = 19–27 Ω for limits between 50 and 200 Hz; Fig. S5). The deviation of the parameters at lower frequencies is ascribed to the dominance of the film response. The Randles + film circuit was therefore used for CdS/rGO/GCE, as a better model that accounts for the behavior across the whole frequency spectrum.

The fitted solution resistance R_s is similar for the three electrodes (84–91 Ω), as expected for the same cell and electrolyte. Independent of any equivalent circuit, the measured impedance modulus of CdS/rGO/GCE is 36–67 times lower than that of the bare GCE between 0.1 and 1 Hz (Fig. S4).

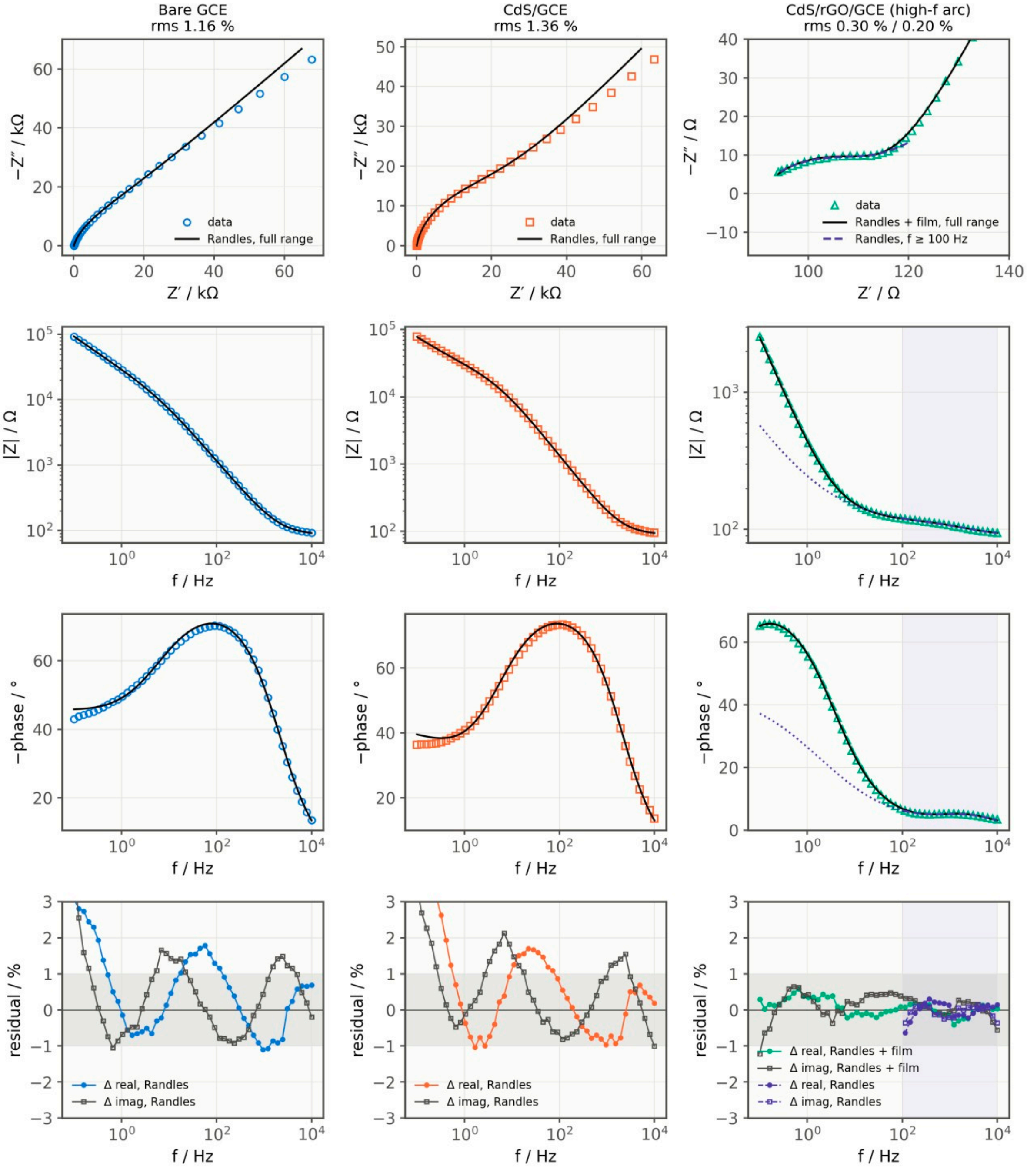

Fig. S3. Equivalent-circuit fits the EIS spectra of the bare GCE (left), CdS/GCE (center), and CdS/rGO/GCE (right) in 5 mM $[Fe(CN)_6]^{3-/4-}$ at 0.2 V vs. Ag/AgCl. Rows, from top: Nyquist plot, impedance modulus, phase angle, and relative residuals (grey band: ±1%). Bare GCE and CdS/GCE: Randles circuit fitted to the full spectrum (solid lines). CdS/rGO/GCE: Randles + film circuit fitted to the full spectrum (solid black) and Randles circuit fitted to f ≥ 100 Hz (dashed; dotted: extrapolation below 100 Hz; shaded: fitted range); the Nyquist panel shows the high-frequency region.

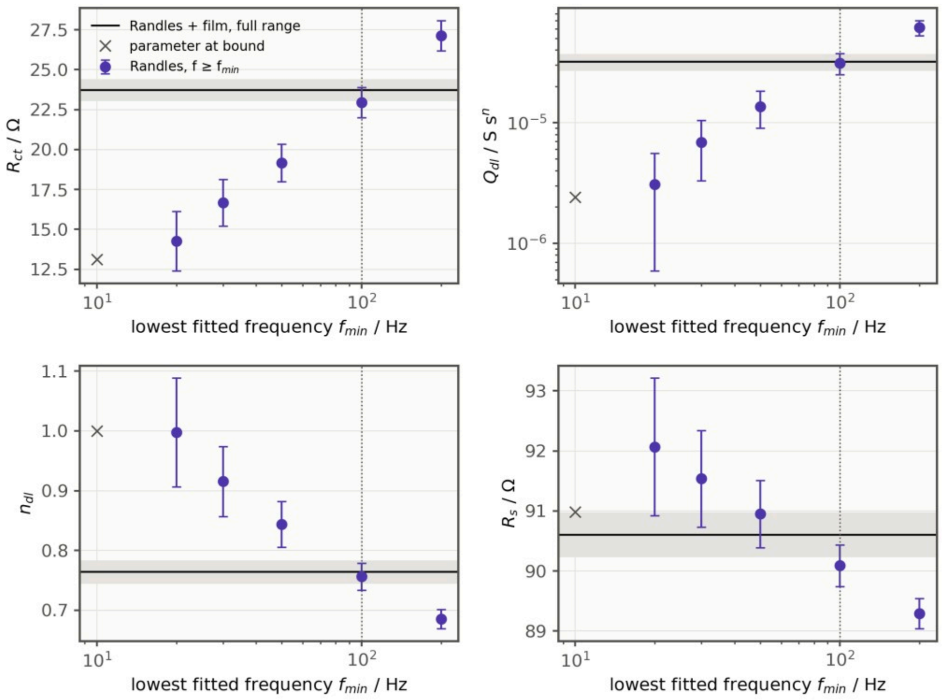

Fig. S4. CdS/rGO/GCE: parameters of the Randles circuit fitted from a lowest frequency f_{min} up to 10 kHz (symbols, ± 1σ), compared with the Randles + film circuit fitted to the full spectrum (line, ± 1σ band). ×: parameter at its bound (fit rejected). Dotted line: cut-off used in Table S5 (100 Hz).

Table S5. Fitted equivalent-circuit parameters (± 1σ) and rms deviations of the EIS spectra.

Parameter	Bare GCE Randles	CdS/GCE Randles	CdS/rGO/GCE Randles + film	CdS/rGO/GCE Randles, f ≥ 100 Hz
Fit range	0.1 Hz – 10 kHz	0.1 Hz – 10 kHz	0.1 Hz – 10 kHz	100 Hz – 10 kHz
R_s / Ω	84.1 ± 0.5	88.3 ± 0.5	90.6 ± 0.4	90.1 ± 0.3
R_{ct} / Ω	(1.81 ± 0.05) × 10^4	(2.15 ± 0.04) × 10^4	23.7 ± 0.7	22.9 ± 0.9
Q_{dl} / S s^n	(3.28 ± 0.04) × 10^{-6}	(2.34 ± 0.03) × 10^{-6}	(3.2 ± 0.5) × 10^{-5}	(3.1 ± 0.6) × 10^{-5}
n_{dl}	0.8640 ± 0.0018	0.8950 ± 0.0017	0.764 ± 0.019	0.76 ± 0.02
σ / Ω $s^{-1/2}$	(5.50 ± 0.04) × 10^4	(3.83 ± 0.04) × 10^4	156 ± 5	276 ± 7
R_f / Ω	–	–	(1.59 ± 0.05) × 10^4	–
Q_f / S s^n	–	–	(6.46 ± 0.02) × 10^{-4}	–
n_f	–	–	0.869 ± 0.003	–
rms deviation / %	1.16	1.36	0.30	0.20

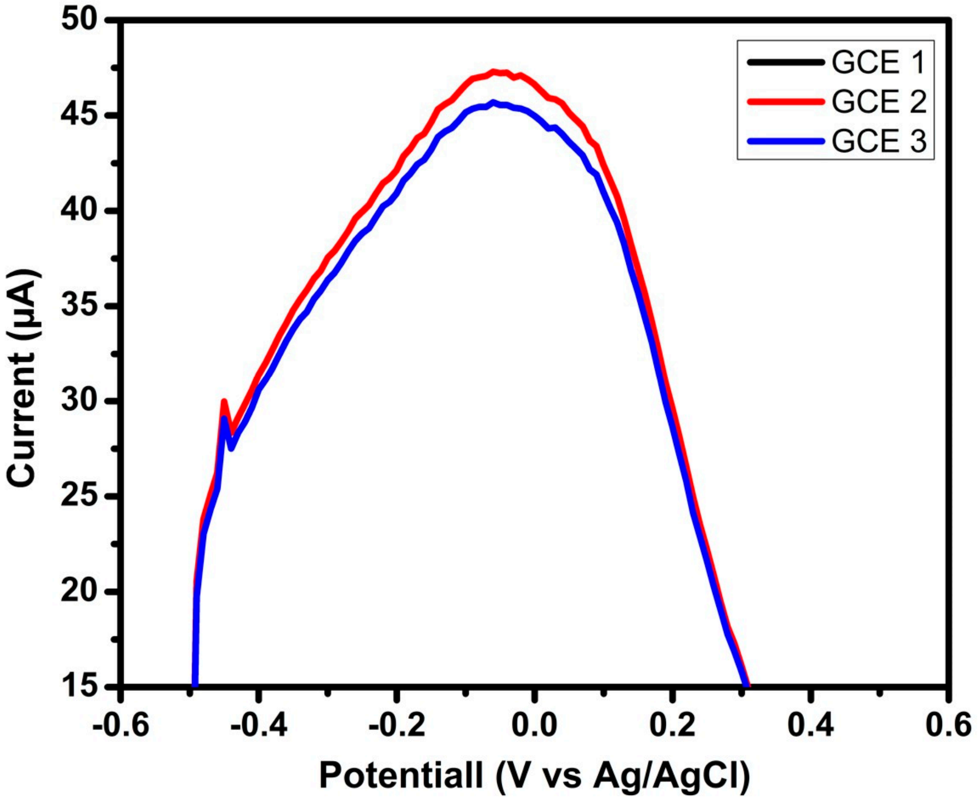

Fig. S5. Raw DPASV for reproducibility of CdS/rGO/GCE for Cu^{2+} sensing

References:

1. Kumar, R.,; Jahan, K.,; Nagarale, R.K.,; Sharma, A., Nongassing Long-Lasting Electro-Osmotic Pump with Polyaniline-Wrapped Aminated Graphene Electrodes. *ACS Appl. Mater. Interfaces* **2015**, *7*, 593–601, doi:10.1021/am506766e.
2. Herrera-Gomez, A.; Guzman-Bucio, D.M.; Carmona-Carmona, A.J.; Cortazar-Martinez, O.; Mayorga-Garay, M.; Cabrera-German, D.; Ospina-Ocampo, C.A.; Crist, B.V.; Raboño-Borbolla, J. Double Lorentzian Lineshape for Asymmetric Peaks in Photoelectron Spectroscopy. *Journal of Vacuum Science & Technology A* **2023**, *41*, 4.
